# Identification of prognostic and diagnostic signatures for cancer and acute myocardial infarction: multi-omics approaches for deciphering heterogeneity to enhance patient management

**DOI:** 10.3389/fphar.2023.1249145

**Published:** 2023-09-14

**Authors:** Na Yuan, Hai-Hua Pan, Yan-Shan Liang, Hui-Lin Hu, Chang-Lin Zhai, Bo Wang

**Affiliations:** ^1^ The First Hospital of Jiaxing Affiliated Hospitial of Jiaxing University, Jiaxing, Zhejiang, China; ^2^ Affiliated Dongguan Hospital, Southern Medical University, Dongguan, Guangdong, China; ^3^ The Second Affiliated Hospital of Jiaxing University, Jiaxing, Zhejiang, China

**Keywords:** signature, AMI, cancer, machine learning, prognosis

## Abstract

Patients diagnosed with cancer face an increased risk of cardiovascular events in the short term, while those experiencing acute myocardial infarction (AMI) have a higher incidence of cancer. Given limitations in clinical resources, identifying shared biomarkers offers a cost-effective approach to risk assessment by minimizing the need for multiple tests and screenings. Hence, it is crucial to identify common biomarkers for both cancer survival and AMI prediction. Our study suggests that monocyte-derived biomarkers, specifically WEE1, PYHIN1, SEC61A2, and HAL, hold potential as predictors for cancer prognosis and AMI. We employed a novel formula to analyze mRNA levels in clinical samples from patients with AMI and cancer, resulting in the development of a new risk score based on expression profiles. By categorizing patients into high-risk and low-risk groups based on the median risk score, we observed significantly poorer overall survival among high-risk patients in cancer cohorts using Kaplan-Meier analysis. Furthermore, calibration curves, decision curve analysis (DCA), and clinical impact curve analyses provided additional evidence supporting the robust diagnostic capacity of the risk score for AMI. Noteworthy is the shared activation of the Notch Signaling pathway, which may shed light on common high-risk factors underlying both AMI and cancer. Additionally, we validated the differential expression of these genes in cell lines and clinical samples, respectively, reinforcing their potential as meaningful biomarkers. In conclusion, our study demonstrates the promise of mRNA levels as biomarkers and emphasizes the significance of further research for validation and refinement.

## Introduction

Acute myocardial infarction (AMI) and cancer are significant contributors to morbidity and mortality globally (Psaty and Vasan, 2023). Despite limited references on the relationship between these two conditions, research indicates that patients diagnosed with cancer are at a higher short-term risk of experiencing cardiovascular events, while those with acute myocardial infarction have an increased incidence of cancer (Rinde et al., 2017; Leening et al., 2023). These potential links imply a latent connection exists between cancer survival and AMI incidence. Therefore, identifying common biomarkers for both cancer survival and AMI prediction is of utmost importance.

The observation that cancer patients face an elevated risk of cardiovascular events is a matter of concern. Studies have shown that cancer survivors experience cardiac complications, such as myocardial infarction, heart failure, and arrhythmias, at rates higher than the general population (Howard et al., 2019). It is hypothesized that this increased risk is multifactorial, involving both direct effects of cancer treatment (e.g., chemotherapy-induced cardiotoxicity) and shared risk factors between cancer and cardiovascular diseases (Shaikh and Shih, 2012). For instance, inflammation, oxidative stress, and endothelial dysfunction, which are common processes in both cancer and cardiovascular diseases, may contribute to the development of adverse cardiac events in cancer patients (Libby and Kobold, 2019). Therefore, identifying common biomarkers that can predict both cancer survival and AMI might help identify patients at higher risk for cardiovascular complications during cancer treatment.

On the other hand, the association between AMI and the subsequent occurrence of cancer has also been documented. Multiple studies have demonstrated an increased incidence of various types of cancer, including lung, colorectal, and hematological malignancies, among individuals with a history of AMI (Leening et al., 2023). Given the potential bidirectional relationship between AMI and cancer, it is vital to explore common biomarkers that might aid in early detection and improve patient outcomes for both conditions. In recent years, advances in molecular profiling technologies have paved the way for the discovery of potential shared biomarkers for cancer survival and AMI prediction (Zhao et al., 2022). Transcriptomics has emerged as a powerful tool for comprehensively analyzing gene expression patterns and identifying differentially expressed genes associated with both diseases (Wang et al., 2009; Ding et al., 2022). Integration of multi-omics data, combining transcriptomics with other molecular profiling techniques such as genomics and proteomics (Reel et al., 2021), holds even greater potential in unraveling the complex interplay between cancer and AMI. In our study, we used ovarian cancer (OC) samples to explore the common diagnostic and prognostic signature of AMI may seem unconventional at first. However, OC and AMI might share certain risk factors, such as obesity, diabetes, and smoking, which can predispose individuals to both conditions (Stewart et al., 2019; Kuhn et al., 2020). In this study, we used OC samples as an example. By studying OC samples, we can investigate if there are common molecular pathways or biomarkers (Aydin et al., 2019) associated with these shared pathways that might contribute to the development of both OC and AMI.

In our study, we applied a novel formula to analyze mRNA levels in clinical samples from AMI and OC. The calculation resulted in the generation of a new risk score based on the expression profiles. The utilization of risk scores demonstrates the ability to accurately predict the probability of AMI incidence and the prognosis of OC patients. Most important of all, these findings demonstrate the potential of utilizing shared biomarkers (WEE1, PYHIN1, SEC61A2, and HAL) to predict outcomes in both cancer and AMI patients.

## Materials and methods

### Pre-processing of bulk transcriptome data

For the AMI cohort, two independent datasets were analyzed on the GPL6244 platform, namely, GSE59867 (111 AMI patients and 46 stable CAD patients at admission) and GSE62646 (28 AMI patients and 14 stable CAD patients at admission) (Pan et al., 2023). The peripheral blood cohort of OC was obtained from GSE31682, which comprises 20 healthy controls and 48 OC patients. After excluding patients with incomplete follow-up information (Feng et al., 2022), we obtained RNA sequencing (RNA-seq) data from both the Cancer Genome Atlas (TCGA) database (Blum et al., 2018) and the International Cancer Genome Consortium (ICGC) database. Additionally, data from the Gene Expression Omnibus (GEO) database (Barrett et al., 2013) was obtained for the GPL570 platform (*n* = 597), which included GSE19829, GSE18520, GSE9891, GSE26193, GSE30161, and GSE63885. To integrate the ICGA and TCGA data and define the meta-RNA-seq dataset, the meta-microarray dataset was defined using the GPL570 platform. Finally, the “sva” package was utilized to effectively address and eliminate batch effects across the different datasets.

### Pre-processing of single-cell RNA sequencing data

Considering the limited availability of human AMI single-cell RNA (scRNA) sequencing datasets, we employed a mouse single-cell sequencing dataset (GSE135310) as an alternative (Pan et al., 2023). This dataset includes single-cell RNA sequencing files for cardiac CD45^+^ total leukocytes, isolated from mice subjected to AMI or sham surgery at various time points. As we lacked peripheral blood single-cell data from healthy individuals within the same batch, we focused on analyzing datasets obtained before and after chemotherapy from the same batch. In this regard, we obtained the GSE213243 dataset, comprising 2 peripheral blood samples from OC patients. In summary, we performed a series of data filtering steps to ensure the quality of our scRNA data. We retained cells with an expression of RNA features ranging from 200 to 2500 while keeping the percentage of mitochondrial RNA content below 10%. Additionally, we employed the Harmony algorithm to mitigate batch effects in our analysis. To annotate all clusters, we utilized the “SingleR” package for comprehensive annotation.

### Clinical samples

As consistent with our previous publication (Pan et al., 2023), we performed mRNA validation of peripheral blood samples using the same cohort of patients. In brief, ten early AMI patients and ten CAD patients were recruited from our hospital between January 2023 and March 2023. Blood samples were collected from the patients shortly after experiencing chest pain, before the administration of antiplatelet or anticoagulant drugs. Peripheral blood mononuclear cells (PBMCs) were isolated from the collected blood samples using established techniques (Boyum, , 1968).

### Immunohistochemical techniques and RT-qPCR

Immunohistochemistry (IHC) staining involves the use of antibodies that are specifically designed to recognize and bind to target antigens of interest. The antibodies are labeled with a chromogenic or fluorescent dye, enabling the visualization and localization of the target molecules under a microscope. The IHC sections utilized in this study were obtained from the Human Protein Atlas (HPA) database. To validate the mRNA expression levels of AMI, PBMCs from patients were utilized. As for OC, cell lines were employed for the validation of mRNA expression levels. IOSE-80 (CP-H055), and SKOV3 (CL-0215) were purchased from Procell Life Science and Technology Co. Ltd. They were cultured in RPMI 1640 medium supplemented with 10% FBS and 1% penicillin/streptomycin at 37°C in a humidified incubator with 5% CO_2_. In short, total RNA was extracted from samples using the FastPure Cell/Tissue Total RNA Isolation Kit V2 (Vazyme, Nanjing, China). Subsequently, RT-qPCR was performed on a LightCycler 480 II Real-time PCR instrument with the HiScript III All-in-one RT SuperMix Perfect for qPCR (Vazyme, Nanjing, China) and ChamQ universal SYBR qPCR Master Mix (Vazyme, Nanjing, China). We used the 2^^-∆∆Ct^ method to calculate gene expression levels. The primer sequences used were designed based on previously published references and PrimerBank database (Wang et al., 2012), including WEE1 (Ma et al., 2022), PYHIN1 (Lee et al., 2019), SEC61A2, and HAL (Kozaczek et al., 2019).

## CIBERSORT

CIBERSORT is a widely used computational tool for analyzing gene expression data and estimating the relative abundance of immune cell populations within complex tissue samples (Chen et al., 2018). It utilizes a deconvolution algorithm to infer cell type proportions from bulk RNA sequencing data. We used the mRNA expression profile data from GSE59867, GSE31682, and meta-RNA-seq datasets (TCGA-OV and ICGC-OV) as inputs. The algorithm utilizes a support vector regression model trained on the signature matrix to determine the relative abundance of each cell type.

### Weighted gene co-expression network analysis

In our study, we utilized the Weighted Gene Co-expression Network Analysis (WGCNA) (Langfelder and Horvath, 2008) in order to investigate gene expression patterns and identify gene modules with similar expression profiles. WGCNA is a powerful tool that allows us to construct a co-expression network based on correlations between genes across different samples. By analyzing the module and module-trait (monocyte score from CIBERSORT) relationships, we can uncover biologically relevant modules associated with specific traits or phenotypes of interest. We established the optimal soft thresholding powers (β) for OC and AMI samples to be *β* = 7 and *β* = 9, respectively. Additionally, we ensured that each module consisted of no fewer than 50 genes.

### Least absolute shrinkage and selection operator regression

We used cross-validation (10-flod) to determine the optimal value for λ. In short, our objective was to determine the optimal model λ value by constructing a penalty function with the occurrence of AMI as the endpoint event and the variation in gene expression as the variable for each sample. The Least absolute shrinkage and selection operator (LASSO) regression model is fit on the training set for each λ value (Cheng et al., 2022), and the performance is evaluated on the validation set using a chosen metric, such as mean squared error or area under the curve. The λ value that minimizes the error on the validation set is considered the optimal choice. Hence, we conducted a more comprehensive screening of potential diagnostic genes from the pool of monocyte-associated genes.

### Establishment and validation of the logstic regression model

We utilized a nomogram based on Logistic regression to facilitate predictive modeling and risk assessment (Zhang et al., 2022). By fitting the logistic regression equation, we estimated the coefficients of the four variables (WEE1, PYHIN1, SEC61A2, and HAL) and captured their contributions to the probability of the outcome. Each predictor variable was assigned a corresponding point value based on its coefficient. The total points were summed up to determine the individual’s predicted probability of the outcome. Moreover, we utilized calibration curves, decision curve analysis (DCA) curves, and clinical impact curves to validate the performance of the nomogram in GSE62646 and GSE59867.

### Establishment and validation of the Cox regression model

The Cox regression model, also referred to as the proportional hazards model, is a widely employed statistical approach utilized in survival analysis to analyze time-to-event data. To construct the Cox regression model, we initially selected a group of potential predictor variables, namely, WEE1, PYHIN1, SEC61A2, and HAL. Subsequently, we performed model training by fitting the Cox regression equation to the meta-RNA-seq cohorts, thereby estimating the coefficients for each gene. The risk score was then calculated using the formula: risk score = Σ (Exp_i_ * coef_i_), where coef and Exp represent the coefficient and expression of each gene, respectively. After establishing the Cox regression model, we proceeded to validate its performance utilizing meta-microarray cohorts. We employed calibration plots, receiver operator characteristic (ROC) curves, and log-rank tests to assess the model’s efficacy in distinguishing between high-risk and low-risk individuals.

### Enrichment analysis

We employed hallmark gene sets (h.all.v7.5.1.symbols.gmt) (Liberzon et al., 2015), which are collections of genes representing key biological processes and signaling pathways. These gene sets cover a wide range of fundamental cellular activities, such as cell cycle regulation, DNA repair, immune response, and metabolism. By applying Gene Set Variation Analysis (GSVA) (Hanzelmann et al., 2013), we transformed our gene expression data into pathway enrichment scores for each sample. This was achieved by comparing the expression levels of genes within each hallmark gene set to the background distribution in our dataset. The resulting enrichment scores provided quantitative measures of the activity levels of these pathways in individual samples.

### Statistical analysis

Statistical analysis was performed using R software (v4.1.2). To evaluate differences, the significance of most cases was assessed using the Wilcoxon rank-sum test. Statistical significance was defined as a *p*-value below 0.05 and indicated as *p < 0.05, **p < 0.01, or ***p < 0.001.

## Results

### Monocytes may serve as indicators for predicting AMI and the prognosis of cancer

**FIGURE 1:**
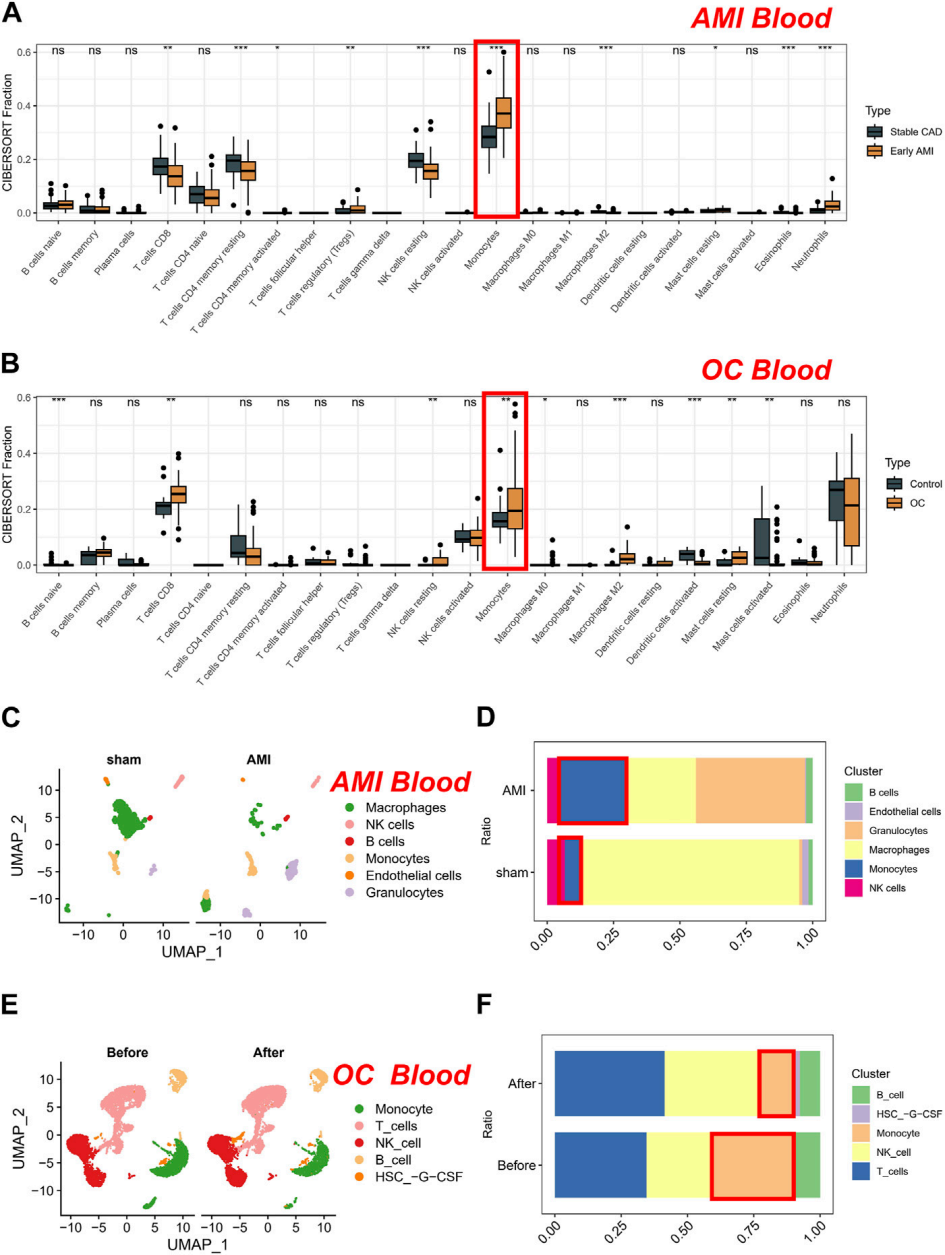
Analysis of cell proportions in AMI and CAD patients and OC patients. **(A)** Comparison of cell proportions using the CIBERSORT algorithm in peripheral blood microarray dataset of AMI and CAD patients. **(B)** Comparison of cell proportions using the CIBERSORT algorithm in peripheral blood microarray dataset of OC patients compared to normal individuals. **(C)** Dimensionality reduction analysis on single-cell data from sham and AMI. **(D)** The proportion of cells in sham and AMI groups. **(E)** Dimensionality reduction analysis on single-cell data from OC patients. **(F)** The proportion of cells in OC groups (Before and after chemotherapy).

Coronary artery disease (CAD) is a principal cause of morbidity and mortality worldwide. Patients with stable CAD are still at risk of AMI, which is a severe complication of CAD. Thus, in clinical settings, patients with CAD are commonly used as control groups to investigate changes in blood indicators among AMI patients. In our study, we first compared differences in cell proportions using the CIBERSORT algorithm on a peripheral blood microarray dataset consisting of AMI and CAD patients. AMI patients exhibit decreased levels of CD8^+^ T cells, memory CD4^+^ T cells, resting NK cells, and M2-type macrophages. Conversely, they have increased levels of Tregs, resting mast cells, neutrophils, and monocytes (Figure 1A). Subsequently, we further investigated the changes in cell content in the peripheral blood of OC patients compared to normal individuals. Interestingly, only the trend of monocytes cell changes was consistent with that of AMI patients. This suggests a potential common role of monocytes in both conditions (Figure 1B). To further validate the robustness of our results, we conducted dimensionality reduction analysis on single-cell data from both the sham and AMI patients, resulting in six distinct clusters that could be clearly distinguished: monocytes, endothelial cells, macrophages, granulocytes, NK cells, and B cells (Figure 1C). Of note, after AMI occurred, the proportion of monocytes increased, consistent with the results of bulk transcriptome analysis. Due to the lack of peripheral blood single-cell data from healthy individuals from the same batch, we analyzed the datasets obtained before and after chemotherapy from the same batch. Similarly, the single-cell data from patients were sorted into four clusters (Figure 1D), with a significant decrease in the proportion of monocytes observed after chemotherapy. Therefore, the proportion of monocytes in the peripheral blood of patients decreased when the tumor-load was reduced by chemotherapy. Conversely, monocytes proportion significantly increased when the tumor occurred (Figure 1E). Given that pathology is often considered the gold standard for cancer diagnosis, we re-assessed the prognostic value of monocytes (CD14 is a typical marker for monocytes) in the TCGA-OV cohort (cancer tissues). Our results also demonstrate that monocytes are a risk factor for OC prognosis. Specifically, as the expression of CD14 increases in bulk tissues, the prognosis of patients worsens (Supplementary Figure S1).

### Biomarkers derived from monocytes using WGCNA in AMI and cancer

**FIGURE 2:**
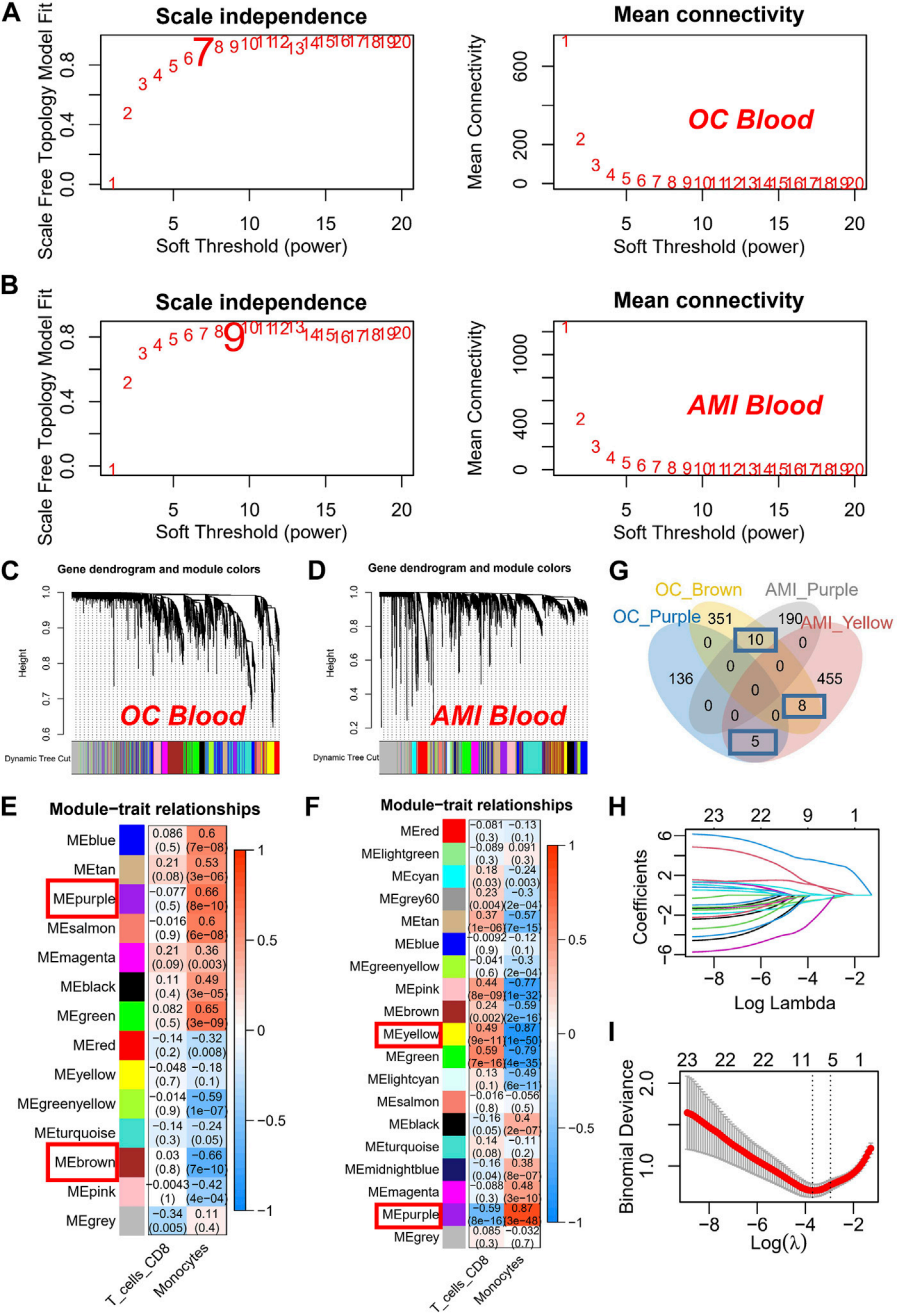
Construction and Analysis of Co-expression Networks for Monocyte-Related Genes. **(A)** Determination of soft threshold powers (β) for OC samples in the co-expression network construction. **(B)** Determination of soft threshold powers (β) for AMI samples in the co-expression network construction. **(C)** Clustering dendrograms, with dissimilarity based on topological overlap, together with assigned module colors in OC datasets. **(D)** Clustering dendrograms, with dissimilarity based on topological overlap, together with assigned module colors in AMI datasets. **(E)** Correlation coefficients and *p*-values used to identify key modules most correlated with the monocyte score in OC datasets. **(F)** Correlation coefficients and *p*-values used to identify key modules most correlated with the monocyte score in AMI datasets. **(G)** Venn plot of key modules. **(H)** The gene signature selection of optimal parameter (lambda). **(I)** LASSO coefficient profiles genes were selected by the optimal lambda.

To enhance gene filtration from monocytes, we integrated the entire gene expression profile into WGCNA. Additionally, we utilized the score of monocyte expression in the CIBERSORT algorithm as a clinical feature to identify key modules most pertinent to monocyte expression. During the construction of the co-expression network, we observed optimal soft threshold powers of *β* = 7 for OC (Figure 2A) and *β* = 9 for AMI samples (Figure 2B). Through careful examination of correlation coefficients and *p*-values (Figures 2C, D), we determined that the purple and brown modules exhibited the strongest absolute correlation with the monocyte score in OC (Figure 2E), while in AMI, the purple and yellow modules displayed the strongest absolute correlation (Figure 2F). As a result, we designated these four modules as key modules and subsequently identified 23 overlapping genes within them (Figure 2G). Subsequently, we conducted further screening of the aforementioned 23 genes in the AMI dataset using the LASSO algorithm. Our objective was to determine the optimal model λ value by constructing a penalty function with the occurrence of AMI as the endpoint event and the variation in gene expression as the variable for each sample (Figures 2H, I). Consequently, we identified seven genes: HRH4, LTBR, WEE1, HAL, PYHIN1, S100A12, and SEC61A2. Of particular significance, based on these seven genes, we proceeded with prognostic validation utilizing the RNA-seq cohort of OC. Our findings indicated that WEE1, PYHIN1, and SEC61A2 served as risk factors impacting the prognosis of ovarian cancer, while HAL demonstrated a protective effect (Supplementary Figure S2).

In summary, our findings suggest that WEE1, PYHIN1, SEC61A2, and HAL derived from monocytes may serve as a potential predictor for AMI and cancer prognosis.

### Establishment of the monocytes-related diagnostic signature in AMI

**FIGURE 3:**
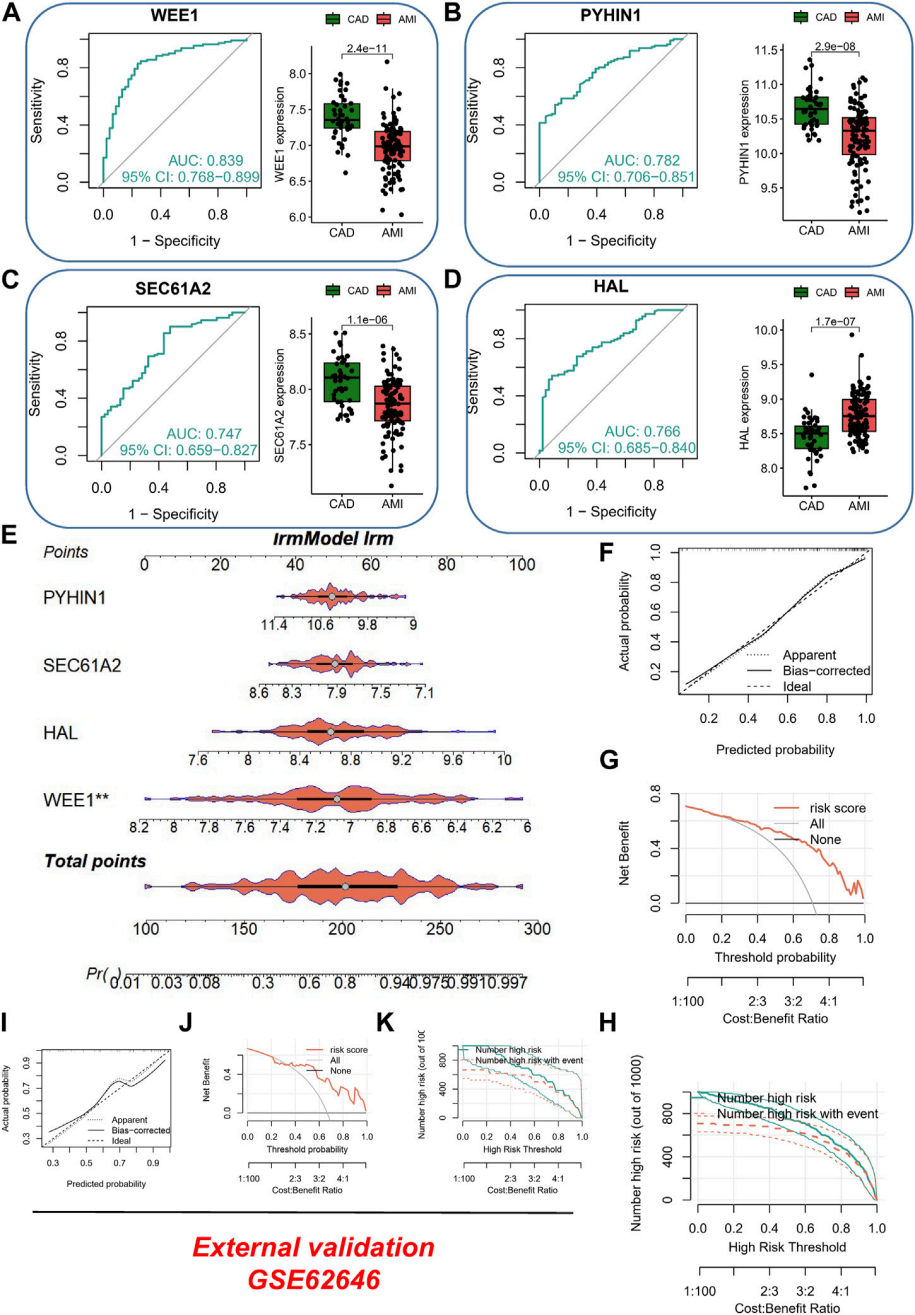
Evaluation of Diagnostic Performance and Clinical Application of the AMI Nomogram. **(A)** ROC curve and box plot of differential expression of WEE1 in different samples. **(B)** ROC curve and box plot of differential expression of PYHIN1 in different samples. **(C)** ROC curve and box plot of differential expression of SEC61A2 in different samples. **(D)** ROC curve and box plot of differential expression of HAL in different samples. **(E)** Development of a nomogram utilizing WEE1, PYHIN1, SEC61A2, and HAL as predictors for AMI diagnosis. **(F)** Calibration curve showing the accuracy and reliability of the nomogram in diagnosing AMI among CAD patients. **(G)** DCA curve demonstrating the diagnostic capacity of the nomogram. **(H)** Clinical impact curve illustrating the robustness of the nomogram for AMI diagnosis. **(I)** External validation of the nomogram using calibration curve. **(J)** External validation of the nomogram using DCA curve. **(K)** External validation of the nomogram using clinical impact curve.

In the AMI dataset, we made noteworthy observations. Firstly, WEE1 (*p* = 2.4e-11) exhibited significant downregulation and displayed superior diagnostic potential, as evidenced by an AUC of 0.839 (95% CI: 0.768–0.899), as shown in Figure 3A. Similarly, PYHIN1 (Figure 3B) and SEC61A2 (Figure 3C) were markedly downregulated in AMI samples and demonstrated favorable diagnostic performance, with AUCs of 0.782 and 0.747, respectively. It is worth mentioning that HAL exhibited significant upregulation in AMI samples, yielding an AUC of 0.766 (Figure 3D). To further enhance the diagnostic accuracy of the model and facilitate its clinical application, we developed a nomogram utilizing the aforementioned four genes as predictors for AMI (Figure 3E). The calibration curve demonstrated the ability of the nomogram to accurately and reliably diagnose AMI among CAD patients (Figure 3F). Moreover, the DCA curve (Figure 3G) and decision curve analysis (Figure 3H) provided further evidence of its robust diagnostic capacity. Importantly, in the validation dataset (GSE62646), the calibration curve (Figure 3I), DCA curve (Figure 3J), and clinical impact curve (Figure 3K) consistently underscored the excellent external validation capability of the nomogram.

### Establishment of the monocytes-related prognostic signature in cancer

**FIGURE 4:**
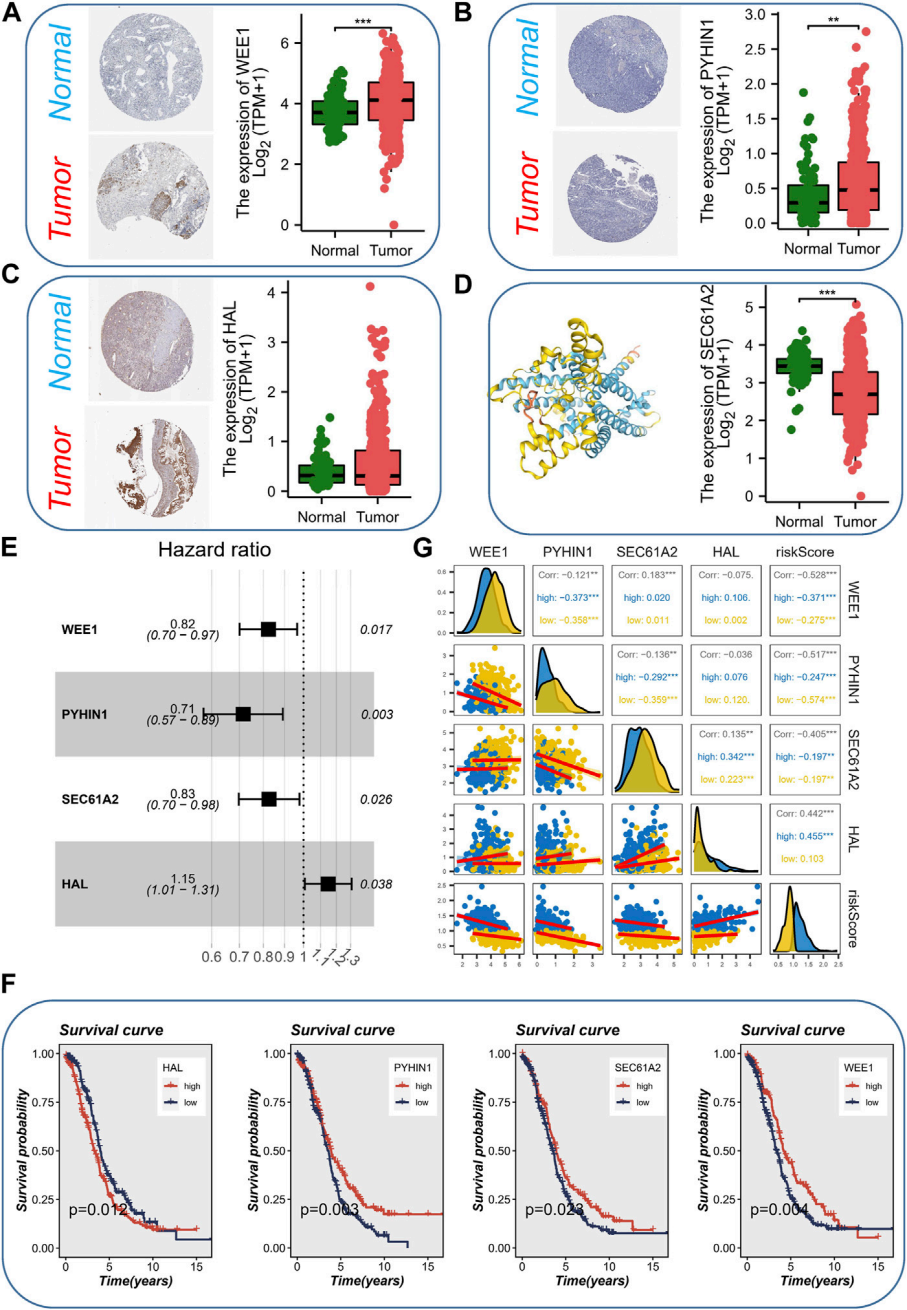
Expression Analysis and Prognostic Model Development. **(A)** The expression level (protein and mRNA) of WEE1 in different samples levels. **(B)** The expression level (protein and mRNA) of PYHIN1 in different samples levels. **(C)** The expression level (protein and mRNA) of HAL in different samples levels. **(D)** The expression level (mRNA) protein structure and of SEC61A2 in different samples levels. **(E)** Development of a risk model using a multi-factorial Cox formula based on the four genes (WEE1, PYHIN1, SEC61A2, and HAL). **(F)** Survival curves showing the impact of each gene in the model on overall survival. **(G)** Correlations among the genes involved in risk model.

**FIGURE 5:**
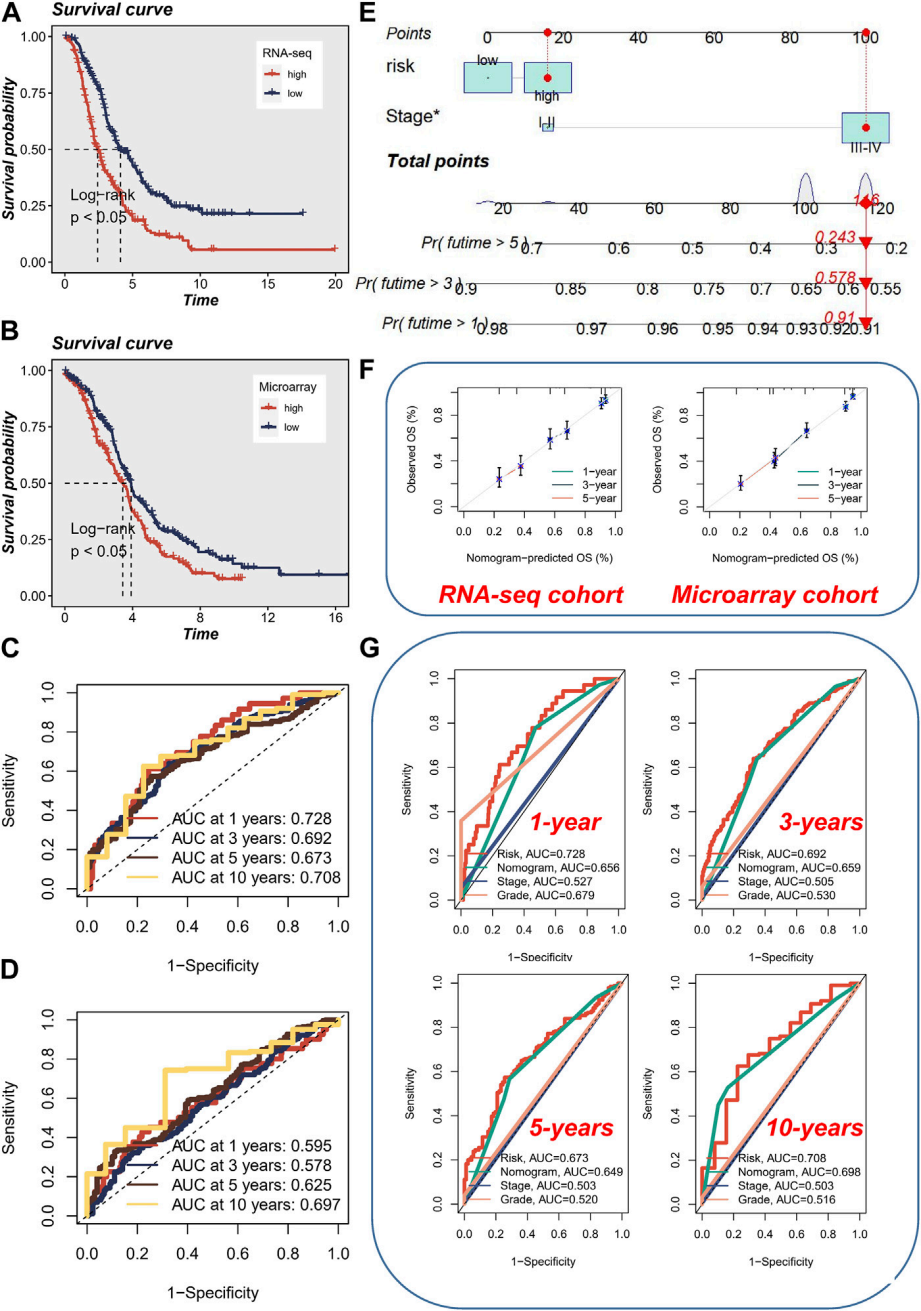
Prognostic Analysis and Nomogram Evaluation. **(A)** Kaplan-Meier survival analysis in the meta-RNA-seq cohort. **(B)** Kaplan-Meier survival analysis in the meta-microarray cohort. **(C)** Evaluation of prognostic accuracy using ROC curves for 1, 3, 5, and 10-year overall survival in the meta-RNA-seq cohort. **(D)** Evaluation of prognostic accuracy using ROC curves for 1, 3, 5, and 10-year overall survival in the meta-microarray cohort. **(E)** Utilization of a visual nomogram integrating FIGO staging and risk stratification to evaluate the risk of ovarian cancer patients. **(F)** Calibration curve of nomogram accuracy in both the meta-RNA-seq cohort and the meta-microarray cohort. **(G)** ROC analysis comparing the performance of the nomogram with other clinical models and risk scores.

Considering that cancer treatment primarily involves surgery and tissue samples are readily available, we conducted a comprehensive investigation into the expression of the aforementioned four genes across various tissue samples by combining the GTEx database and the HPA database. At both the protein and mRNA levels, WEE1 exhibited significantly higher expression in tumor samples (Figure 4A). Similarly, PYHIN1 displayed significantly higher mRNA expression in tumor samples, although no significant protein staining (Figure 4B). In contrast, HAL did not exhibit significant differences in mRNA expression between the two sample types, but protein upregulation was evident in tumor samples (Figure 4C). Unfortunately, an IHC antibody for SEC61A2 was unavailable. However, we characterized the protein’s structure and confirmed its significant upregulation at the mRNA level in normal samples (Figure 4D). Subsequently, to quantify the survival risk for each ovarian cancer patient, we developed a risk model utilizing a multi-factorial Cox formula (Figure 4D) based on the four aforementioned genes (WEE1, PYHIN1, SEC61A2, and HAL). The risk score for each OC patient was calculated using the equation: Risk score = (−0.195 × WEE1 expression) + (−0.335 × PYHIN1 expression) + (−0.191 × SEC61A2 expression) + (0.137 × HAL expression). Subsequently, survival curves were generated for each gene in the model, revealing intriguing findings. PYHIN1, SEC61A2, and WEE1 emerged as protective genes, suggesting that reduced expression of these genes may contribute to prolonged overall survival in patients (Figure 4F). Conversely, HAL was identified as a high-risk gene, implying that increased expression of HAL may be associated with decreased patient survival. Notably, our expression level assessment demonstrated predominantly negative correlations among the genes (Figure 4G). Specifically, HAL displayed negative correlations with WEE1 and PYHIN1, while exhibiting a positive correlation with SEC61A2. Regarding risk scores, a noteworthy positive correlation was solely observed with HAL (*R* = 0.442, *p* < 0.001). The formula described above was applied to both the meta-RNA-seq cohort and the meta-microarray cohort, resulting in the generation of a new risk score based on the expression profile. Patients were then classified into high-risk and low-risk categories using the median risk score. Kaplan-Meier survival analysis conducted in the meta-RNA-seq cohort revealed that high-risk patients exhibited significantly worse overall survival compared to low-risk patients (Figure 5A). Similarly, in the meta-microarray cohort, the high-risk group had a lower likelihood of survival (Figure 5B). To assess the prognostic accuracy of the prognostic features, ROC curves for 1, 3, 5, and 10-year OS were analyzed. In the meta-RNA-seq training cohort, the AUC values for these time points were 0.728, 0.692, 0.673, and 0.708, respectively, (Figure 5C). Similarly, the meta-microarray validation set showed superior AUC values of 0.595, 0.578, 0.625, and 0.697 for the 1, 3, 5, and 10-year AUCs, respectively, (Figure 5D).

To ensure consistency with the AMI risk signature, we utilized a visual nomogram to evaluate the risk of OC patients by integrating FIGO staging and risk stratification (Figure 5E). The accuracy of nomogram was assessed and found to be superior in both the meta-RNA-seq cohort and the meta-microarray cohort (Figure 5F). Additionally, ROC analysis was performed, indicating that early survival prediction using the nomogram outperformed other clinical models and risk scores, whereas for long-term survival prediction (>5 years), utilizing the risk score alone yielded better results (Figure 5G).

### Immune cell infiltration in AMI and cancer

**FIGURE 6:**
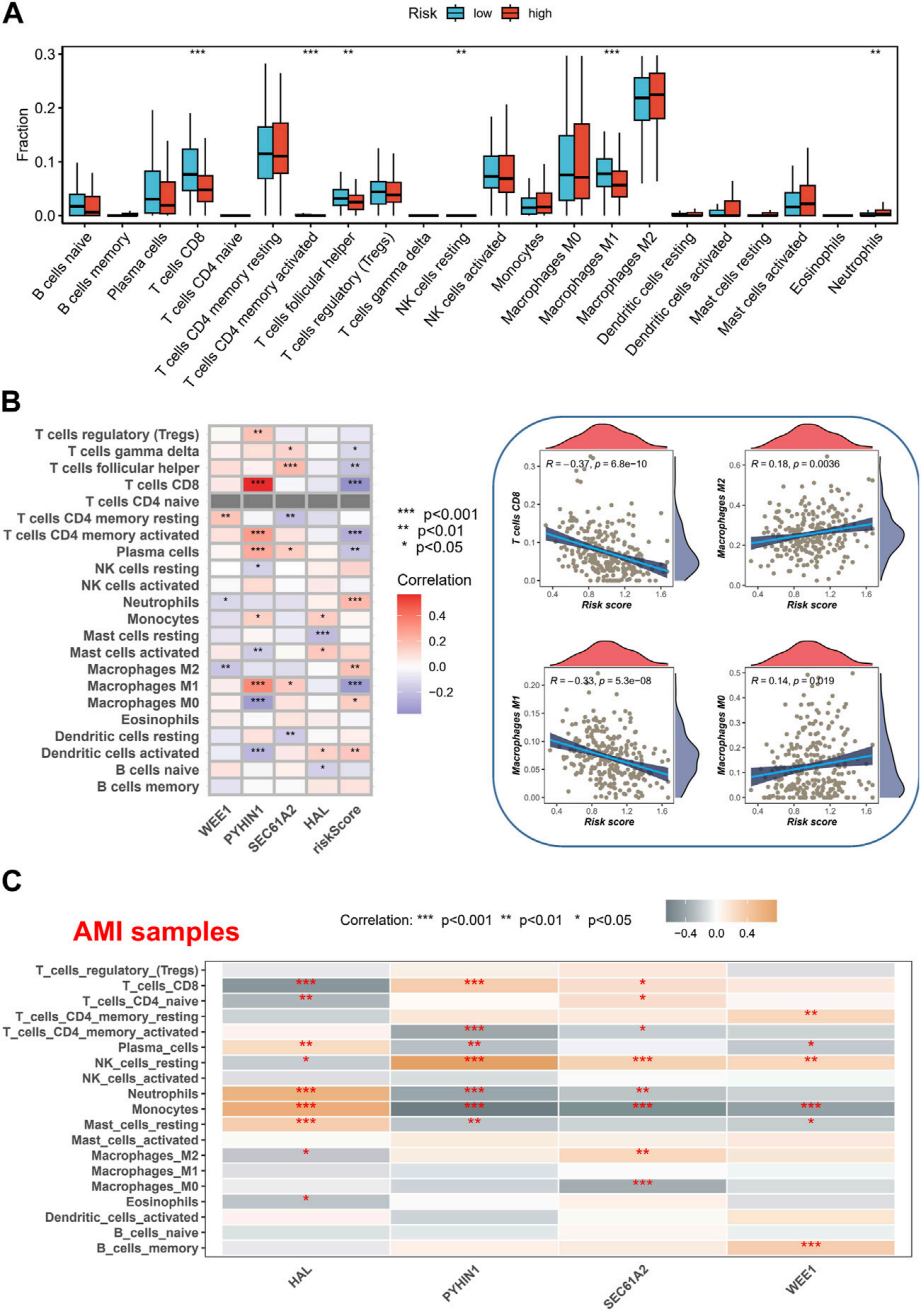
Immune Cell Composition Analysis and Model Gene Correlation. **(A)** Analysis of immune cell composition utilizing the CIBERSORT method in tissue samples of high-risk and low-risk groups. **(B)** Characterization of the correlation between gene expression and immune cell. **(C)** Characterization of the correlation between model genes and immune cells in AMI samples.

We utilized the CIBERSORT method to analyze the immune cell composition of tissue samples, comparing the high-risk and low-risk groups, and associating them with model genes. Interestingly, we discovered that the high-risk group exhibited lower levels of CD8^+^ T cells and M1 macrophages (Figure 6A). CD8^+^ T cells, typically referred to as cytotoxic T lymphocytes, secrete various cytokines involved in immune responses (Mittrucker et al., 2014), while M1-type macrophages are capable of producing pro-inflammatory cytokines (Mills et al., 2016). The reduction of CD8^+^ T cells and M1 macrophages may indicate an “cold environment” in high-risk patients. Furthermore, we observed a significant positive correlation between the expression of the PYHIN1 gene and CD8^+^ T cells, suggesting that the PYHIN1 gene primarily influences changes in the tumor microenvironment by regulating the proliferation of cytotoxic T lymphocytes (Figure 6B). We demonstrated the specific distribution of risk scores and different immune cell types, revealing a negative correlation between CD8^+^ T cells and risk scores. Moreover, it appears that changes in risk scores also influence the proportions of M0/M1/M2 macrophages. Furthermore, we characterized the correlation between model genes and immune cells in AMI samples, which similarly showed a strong significant positive correlation between PYHIN1 gene expression and CD8^+^ T cells, potentially regulating the proliferation of resting NK cells (Figure 6C).

### Enrichment analysis

**FIGURE 7:**
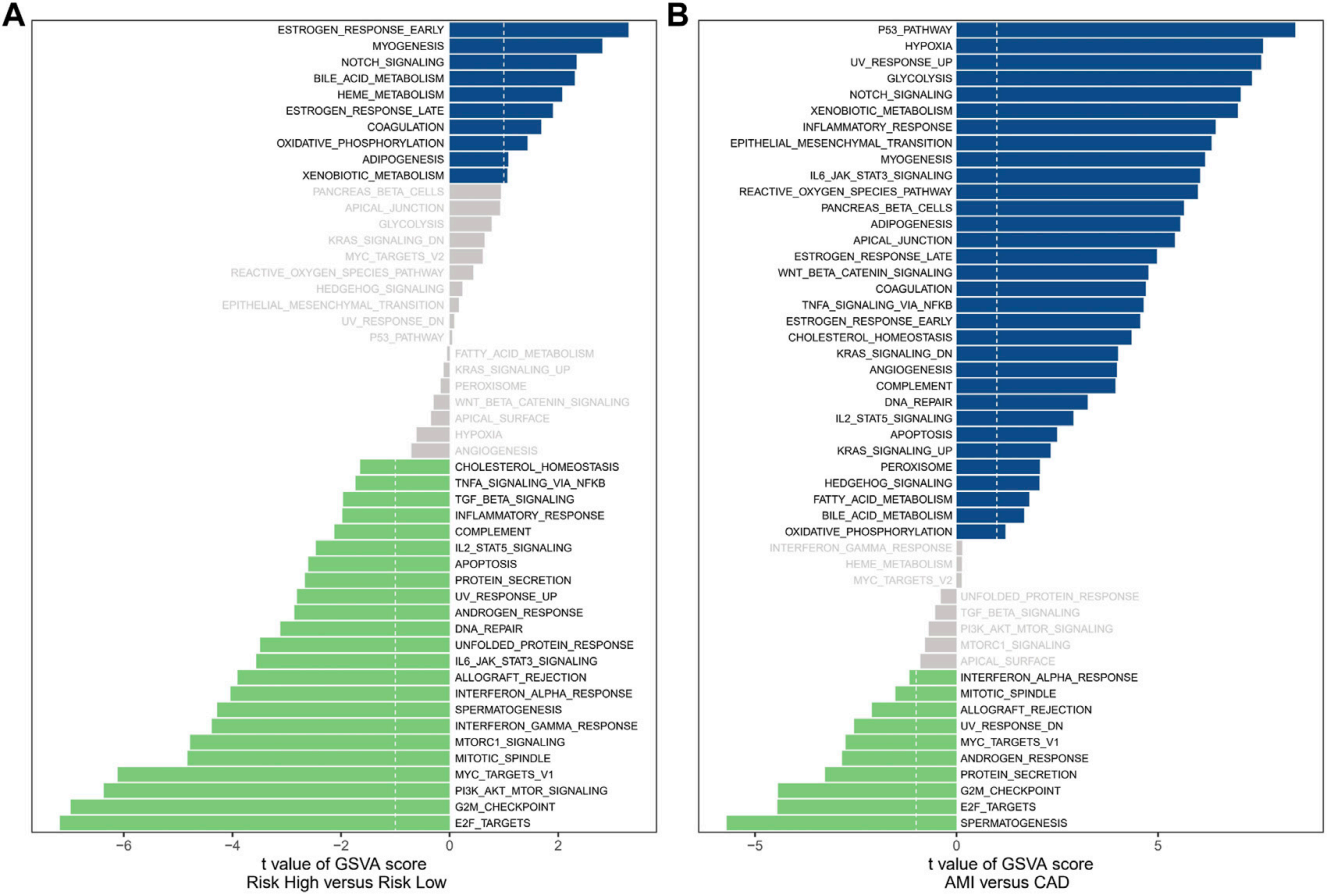
Enrichment analysis. **(A)** The results of Gene Set Variation Analysis in OC samples. **(B)** The results of Gene Set Variation Analysis in AMI samples.

The analysis of hallmark pathway gene features using the GSVA method reveals distinct differences between high-risk and low-risk groups in OC. A direct comparison between these groups demonstrates specific enrichment features. In the high-risk group, the top five enriched features include Estrogen Response Early, Myogenesis, Notch Signaling, Bile Acid Metabolism, and Heme Metabolism (Figure 7A). Conversely, the low-risk group exhibits the top five enriched features: E2F Targets, G2M Checkpoint, PI3K/AKT/MTOR Signaling, MYC Targets V1, and Mitotic Spindle. Additionally, we investigated the significantly different pathway signals between patients with AMI and coronary artery disease. The findings reveal substantial activation of the P53 Pathway, Hypoxia, and Notch Signaling in AMI patients (Figure 7B). Of particular interest is the shared activation of the Notch Signaling pathway, which may provide insight into the common high-risk factors underlying both AMI and cancer.

### Validation of mRNA levels in clinical samples

To validate the reliability of the four prognostic genes identified, qRT-PCR testing was performed on both clinical samples and cell lines. In PBMC samples, we observed consistent differential expression patterns of the four genes with the results obtained from the microarray analysis (Figures 3A–D). Specifically, compared to CAD, WEE1, PYHIN1, and SEC61A2 were downregulated in AMI, while HAL exhibited upregulation (Supplementary Figure S3A). Furthermore, it is noteworthy that the validation performed at the cell lines also demonstrated consistent results with RNA-seq analysis. Specifically, when compared to IOSE-80 cells, SKOV3 cells exhibited upregulation in the expression of WEE1, PYHIN1, and HAL, while the expression of SEC61A2 was downregulated (Supplementary Figure S3B).

## Discussion

Acute myocardial infarction (AMI) and cancer are two prevalent and devastating health conditions that contribute significantly to morbidity and mortality worldwide (Rinde et al., 2017). While extensive research exists on each of these diseases independently, the relationship between AMI and cancer remains relatively understudied. Common biomarkers could help identify patients who are at higher risk of developing either cancer or AMI, enabling tailored preventive strategies. By stratifying individuals based on their risk profiles, healthcare providers can offer targeted interventions, such as lifestyle modifications, pharmacological interventions, and regular surveillance, with the goal of mitigating the risk of both diseases. Moreover, in a clinical setting where resources are often limited, utilizing shared biomarkers may offer a cost-effective approach to risk assessment by reducing the need for multiple tests and screenings.

In our study, our findings suggest that WEE1, PYHIN1, SEC61A2, and HAL derived from monocytes may serve as a potential predictor for AMI and cancer prognosis. WEE1, a protein kinase involved in cell cycle regulation, acts as a critical regulator of the DNA damage response pathway and plays a crucial role in maintaining genomic stability (Okabe et al., 2023; Su et al., 2023). Its overexpression has been observed in various tumor types, contributing to tumor growth, chemoresistance, and poor prognosis. Mohamed et al. (Mohamed et al., 2018) discovered that overexpression of cyclin-dependent kinase 1 (CDK1), CDK4, cyclin B1, and cyclin D1 in adult cardiomyocytes induces stable cell division, leading to significant cardiac regeneration after myocardial infarction. Importantly, they found that inhibition of Wee1, along with Tgf-β, made CDK1 and cyclin B dispensable, highlighting the role of WEE1 as a potential target for promoting cardiomyocyte proliferation. Chen and Gardner (Chen and Gardner, 2004) found that endothelin (ET) promotes proliferation of rat aortic smooth muscle cells by increasing CDK2 and CDC2 activity through the MEK/ERK/RSK signal transduction pathway. They observed that ET treatment led to phosphorylation and inactivation of the inhibitory kinase WEE1, along with upregulation of CDC25A phosphatase, highlighting the role of WEE1 in ET-dependent mitogenesis. PYHIN1, a member of the PYHIN (pyrin and HIN domain-containing) protein family, exerts complex functions ranging from tumor suppression to tumor promotion, depending on the specific tumor (Tong et al., 2019; Ding et al., 2022). Its involvement in DNA damage repair, cell cycle regulation, immune responses, and inflammation contributes to its multifaceted role in cancer progression. de Las Fuentes et al. (2013) investigated the role of SNP-loop diuretic interactions in hypertension across different ethnic groups. In their study on African Americans (AA) and European Americans (EA), they identified several promising loci, including genes such as NUDT12, CHL1, GRIA1, CACNB2, and PYHIN1 for systolic blood pressure (SBP) in AA, and ID3 for diastolic blood pressure (DBP) in AA. These findings suggest that PYHIN1 may play a role in the regulation of blood pressure and response to anti-hypertensive drugs, although no SNP reached genome-wide significance in this small study. Further research in more diverse populations is needed to identify additional variants. SEC61A2, a key component of the SEC61 protein complex, plays a crucial role in protein translocation across the endoplasmic reticulum (ER) membrane (Connerly et al., 2005; Vendrov et al., 2006) investigated NAD(P)H oxidase-mediated signaling in atherosclerosis and identified several genes regulated by thrombin-induced NAD(P)H oxidase, including SEC61A2, in vascular smooth muscle cells (VSMCs). They demonstrated that NAD(P)H oxidase plays a role in the regulation of CD44 and BMP4-Id signaling pathway, which are implicated in restenosis and atherosclerosis. These findings suggest that SEC61A2 and other genes controlled by NAD(P)H oxidase may have important implications for vascular lesion formation. *Homo sapiens* histidine ammonia-lyase (HAL) is an enzyme involved in the catabolism of histidine. It plays a crucial role in modulating histidine metabolism, which influences the immune response, and angiogenesis (Krzymuska, 1964; Blaeschke et al., 2019) conducted a study on pediatric medulloblastoma, a brain tumor with minimal mutational load and low immunogenicity. Despite this, they identified immunogenic tumor-specific peptides in each patient, including peptides derived from the HAL gene. These findings suggest that even in tumors with low mutational load, specific T-cell immunotherapy targeting neoantigens is feasible and may guide future therapeutic approaches. Yu et al. (2015) conducted a study on the association between genetic variants, histidine levels, and incident coronary heart disease (CHD). They identified three rare loss-of-function (LoF) variants in the HAL gene, which encodes histidine ammonia-lyase, and found that these variants had significant effects on blood histidine levels. Furthermore, high blood histidine levels were associated with a reduced risk of developing CHD, suggesting a potential protective role of histidine in both African Americans and European Americans. By identifying these genes as potential biomarkers for both cancer prognosis and AMI prediction, we aim to shed light on the shared molecular mechanisms underlying these diseases. However, further experimental studies are needed to elucidate the specific roles of WEE1, PYHIN1, SEC61A2, and HAL in disease occurrence, progression, and response to treatment in both cancer and AMI.

Moreover, we also applied a novel formula to analyze mRNA levels in clinical samples from a meta-RNA-seq cohort and a meta-microarray cohort. The calculation resulted in the generation of a new risk score based on the expression profiles. Patients were classified into high-risk and low-risk categories using the median risk score, and Kaplan-Meier survival analysis revealed that high-risk patients exhibited significantly poorer overall survival compared to low-risk patients in both cohorts. These findings demonstrate the potential of utilizing shared biomarkers to predict outcomes in cancer patients. Furthermore, our study assessed the prognostic accuracy of the obtained risk score using ROC curves for various time points of overall survival. In the meta-RNA-seq training cohort, the AUC values demonstrated moderate to good accuracy for predicting 1, 3, 5, and 10-year overall survival. Similarly, in the meta-microarray validation set, the AUC values indicated fair to good accuracy for the same time points. To ensure consistent presentation and enhance clinical utility, we integrated FIGO staging and risk stratification into a visual nomogram. Similarly, we created a nomogram for the diagnosis of AMI patients. The calibration curve demonstrated the ability of the nomogram to accurately and reliably diagnose AMI among CAD patients. Moreover, the DCA curve and decision curve analysis provided further evidence of its robust diagnostic capacity. Importantly, in the validation dataset (GSE62646), the calibration curve, DCA curve, and clinical impact curve consistently underscored the excellent external validation capability of the nomogram.

In the context of cancer, the correlation between the risk score and immune signatures indicates that patients with a higher risk score may have a more dysregulated immune system, which could influence their response to immunotherapy. Immunotherapies, such as immune checkpoint inhibitors, have revolutionized cancer treatment by enhancing the immune system’s ability to recognize and eliminate tumor cells. Therefore, our findings suggest that patients with a higher risk score may be more suitable candidates for immunotherapeutic approaches, as they may have a greater potential to respond to these treatments. Similarly, in the context of AMI, the correlation between the risk score and immune signatures implies that the immune response plays a crucial role in the pathogenesis and progression of cardiac injury. Targeting immune-related pathways involved in AMI may provide new avenues for therapeutic interventions. Modulating the immune response, reducing inflammation, and promoting tissue repair are potential strategies for improving outcomes in patients with AMI. Therefore, understanding the relationship between the risk score and immune signatures can guide the development of novel therapies targeted at modulating the immune response in the context of AMI.

Of particular interest is the shared activation of the Notch Signaling pathway, which may provide insight into the common high-risk factors underlying both AMI and cancer. The role of Notch signaling in cancer is complex and contributes to enhanced tumorigenesis through various mechanisms such as angiogenesis, drug resistance, and epithelial to mesenchymal transition. Inhibiting the Notch pathway has emerged as a promising therapeutic strategy, and studies have shown promising results with Notch inhibitory agents in reducing tumorigenic aggressiveness (Sen and Ghosh, 2023; Yu et al., 2023) investigated the role of Notch signaling in innate lymphoid cells (ILCs) in acute coronary syndrome. The study found that activation of the Notch signaling pathway was associated with a shift from ILC1 to ILC2 subsets in peripheral blood of AMI patients, and inhibiting Notch signaling increased ILC1 frequency and interferon-γ secretion while reducing ILC2 frequency and interleukin-5/interleukin-13 production. These findings suggest that Notch signaling may play a role in regulating ILC subsets in AMI patients. Liu et al. (2019) investigated the role of miR-29b and its effect on myocardial infarction (MI) in rats through the Notch signaling pathway. The study demonstrated that downregulation of miR-29b in the MI group was associated with increased expression of Notch1, DII4, Hesl, and NICD1, suggesting that miR-29b inhibits myocardial fibrosis and cardiac hypertrophy by activating the Notch signaling pathway, providing protection against MI. Matsuda et al. (2014) investigated the impact of Notch signaling on human cardiac stem cells (CSCs) and their therapeutic potential in an AMI rat model. They found that reducing Notch signaling by culturing CSCs at low plating density enhanced their proliferation, multi-differentiation potential, and therapeutic efficacy, highlighting the importance of optimizing culture conditions for CSCs in clinical applications.

One practical application of the common signature is its potential in guiding treatment decisions. By profiling the common signature in individual patients, clinicians could better stratify patients and predict their response to specific therapies. For example, if a patient with cancer or AMI has a dysregulated immune-related common signature, it suggests that they may be more likely to benefit from immunotherapeutic or immune-modulatory interventions. This information can help inform treatment selection and improve personalized medicine approaches. Furthermore, the common signature can also guide the development of novel therapeutic strategies. By targeting the shared dysregulated pathways identified in the common signature, researchers and pharmaceutical companies can develop new drugs or repurpose existing ones to effectively treat both cancer and AMI. This approach could lead to the development of combination therapies that simultaneously target the common dysregulated pathways, potentially improving treatment efficacy and patient outcomes. Additionally, the common signature can have implications for prognosis and risk stratification. By assessing the expression levels or activation states of the common signature genes, clinicians may be able to predict disease progression, recurrence, or complications in both cancer and AMI patients. This information can aid in tailoring surveillance strategies and determining appropriate follow-up care for patients at higher risk.

There are several limitations to consider in this study. Firstly, the sample size of the clinical samples used for mRNA analysis was not clearly stated, which may affect the generalizability and statistical power of the findings. Additionally, the study focused on a specific set of biomarkers derived from monocytes, and it is possible that other relevant biomarkers were not considered. The study also primarily relied on retrospective data analysis, which may introduce biases and limit causal interpretations. Further prospective studies are needed to validate the predictive value of these biomarkers in larger, diverse patient populations. Finally, although the shared activation of the Notch signaling pathway is mentioned as a potential underlying factor, the specific mechanistic links between the identified biomarkers, AMI, and cancer prognosis are not fully explored or elucidated. Future research should aim to investigate these mechanisms in order to better understand the biological significance of these biomarkers.

## Conclusion

In conclusion, the identification of shared biomarkers for cancer survival and AMI prediction represents a critical step toward improving patient care for individuals affected by these conditions. By understanding the underlying pathophysiological mechanisms and implementing personalized preventive strategies, healthcare providers can potentially reduce the burden of both diseases and improve patient outcomes. Our study demonstrates the potential of utilizing mRNA levels as biomarkers and highlights the importance of further research in this area to validate and refine these findings.

## Data Availability

The datasets presented in this study can be found in online repositories. The names of the repository/repositories and accession number(s) can be found in the article/Supplementary Material.

## Supplementary material

SUPPLEMENTARY FIGURE S1 Prognostic value of monocytes assessed in the TCGA-OV cohort.

SUPPLEMENTARY FIGURE S2 Forest plot of univariate cox regression analysis.

SUPPLEMENTARY FIGURE S3 Validation of mRNA levels in clinical samples. **(A)** mRNA expression of WEE1, PYHIN1, SEC61A2, and HAL in clinical samples. **(B)** mRNA expression of WEE1, PYHIN1, SEC61A2, and HAL in cell lines.
