# Supplementary figures and images for: Identification of prognostic and diagnostic signatures for cancer and acute myocardial infarction: multi-omics approaches for deciphering heterogeneity to enhance patient management
Source: Front Pharmacol. 2023 Sep 14;14:1249145. doi: 10.3389/fphar.2023.1249145 (PMC10539594; doi:10.3389/fphar.2023.1249145)

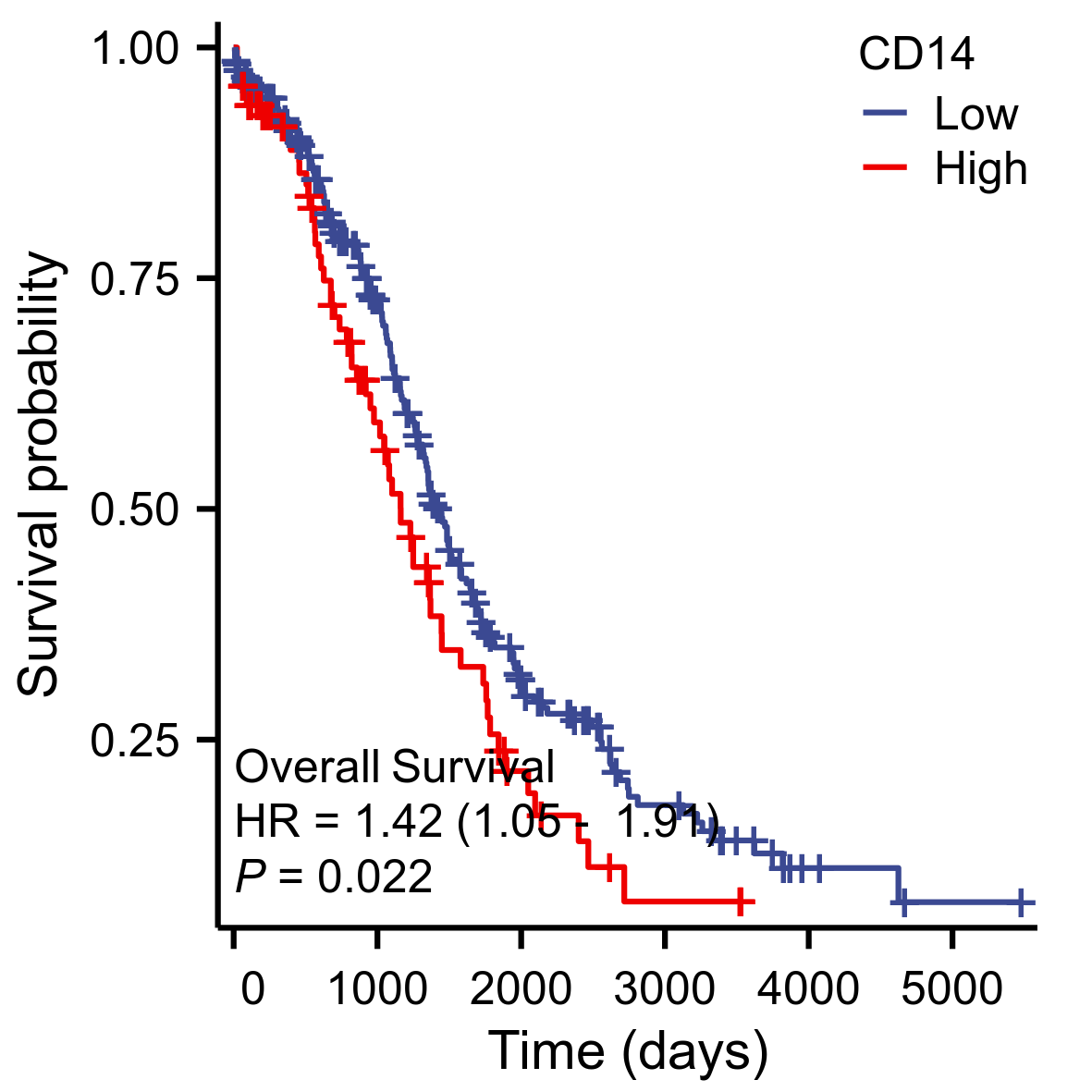

Supplement: Supplementary file 1 [file Image1.TIFF]

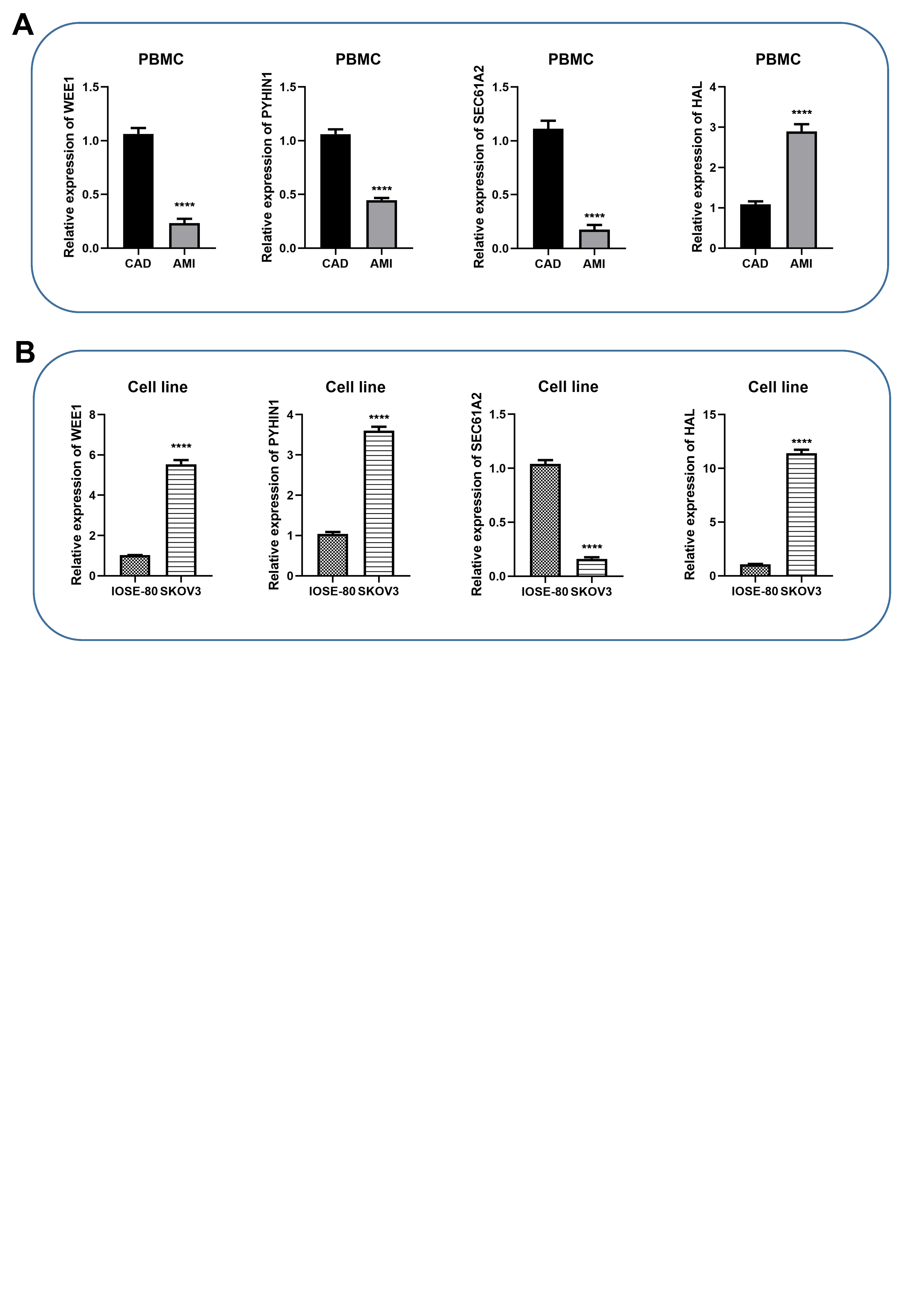

Supplement: Supplementary file 2 [file Image3.TIF]

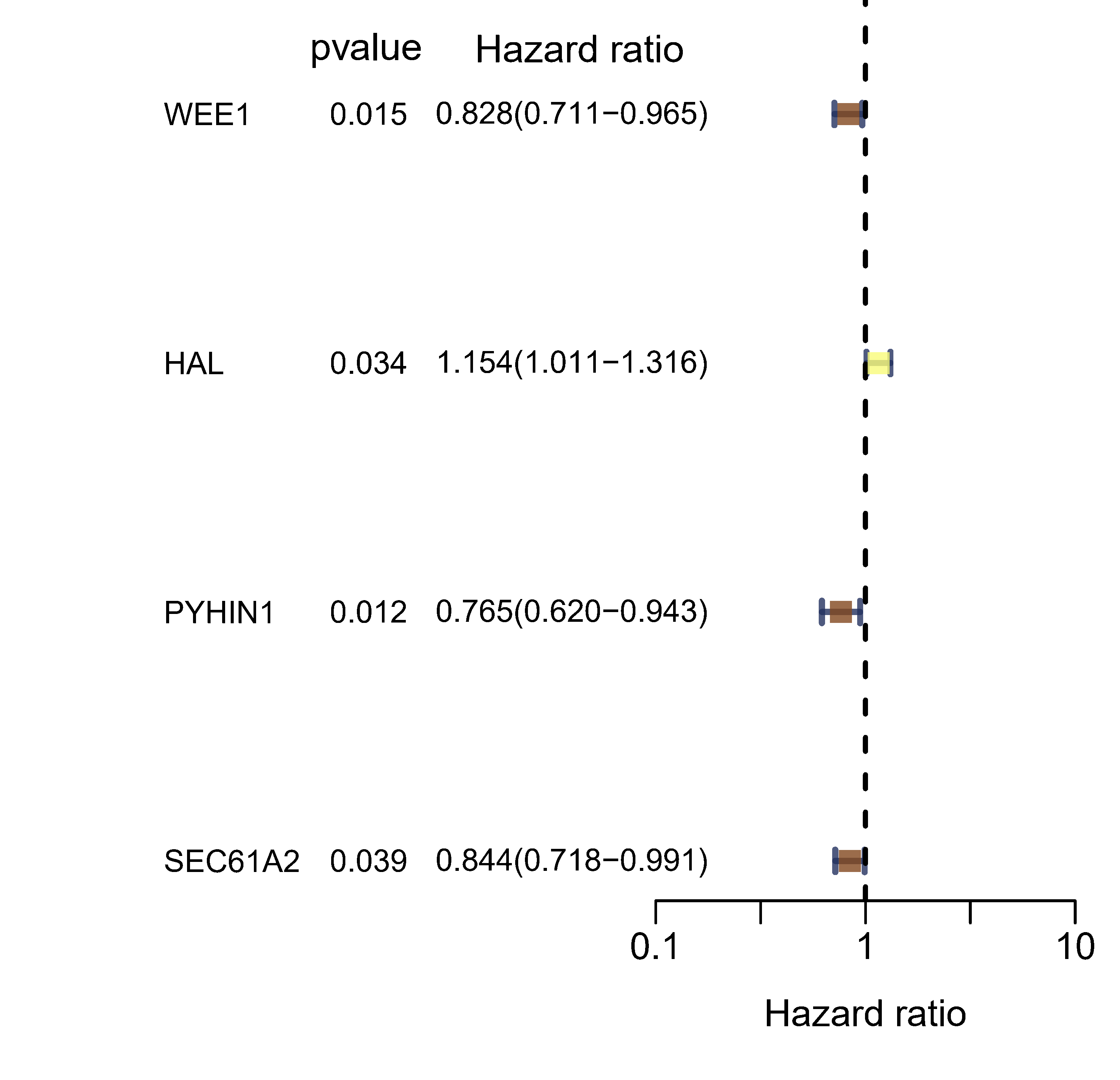

Supplement: Supplementary file 3 [file Image2.TIF]
